# The White Matter Connectome Supporting Speech and Language in the Human

**DOI:** 10.1162/NOL.e.241

**Published:** 2025-12-31

**Authors:** Anthony Steven Dick, Pascale Tremblay

**Affiliations:** Department of Psychology, Florida International University, Miami, FL, USA; Department of Rehabilitation Sciences, Université Laval, Quebec City, QC, Canada

**Keywords:** clinical neuroscience, connectome, language, lateralization, plasticity, speech, white matter

## Abstract

The neurobiology of language has moved beyond the classical dorsal–ventral dichotomy to embrace a more complex, distributed, and dynamic white matter connectome. This special issue, The White Matter Connectome Supporting Speech and Language in the Human, presents 10 empirical studies that leverage traditional and advanced diffusion modeling and analyses—including Restriction Spectrum Imaging, fixel-based analysis, and network control theory—to map this complexity. The contributions are organized into three themes: developmental plasticity, lateralization, and clinical resilience. Findings range from the rapid consolidation of speech categories during sleep and the microstructural scaffolding of early childhood speech, to the surprising stability of reading networks following educational disruption. Novel insights into lateralization challenge binary left–right models, revealing how interhemispheric balance and intrahemispheric asymmetry jointly shape functional dominance. Finally, clinical studies on aphasia, dyslexia, and stroke recovery demonstrate how structural connectivity constrains therapeutic outcomes, identifying specific white matter targets for semantic versus phonological recovery. Collectively, these articles advance a framework where the white matter connectome is not merely a static system, but an active, plastic substrate essential for human communication.

## INTRODUCTION

The study of language neurobiology in the human brain has undergone a paradigm shift over the last two decades. Classical models grounded in the Broca–Wernicke–Geschwind framework emphasized discrete cortical loci connected by a single pathway, the arcuate fasciculus. However, accumulating evidence from modern neuroimaging has dismantled this “localist” view. It is now understood that language emerges from the dynamic interactions of distributed cortical and subcortical systems (Dick & Tremblay, 2012; Duffau, 2008; Schmahmann & Pandya, 2006), scaffolded by a complex architecture of association, projection, and callosal fiber pathways (Dick et al., 2014; Duffau, 2008; Tremblay & Dick, 2016).

This broader network perspective has generated both consensus and controversy. While there is general agreement on the existence of dual dorsal and ventral processing streams, debates persist regarding the precise anatomical definitions of these tracts, their lateralization, and their plasticity in response to experience or injury. Furthermore, the field faces a methodological reckoning: traditional diffusion tensor imaging metrics, while useful (Le Bihan, 2024), often lack the biological specificity to distinguish between crossing fibers, intracellular changes, or complex network topologies. Consequently, prior reviews have called for frameworks that embrace this anatomical complexity, confront methodological limitations through advanced modeling, and account for the substantial individual variability observed in human language function (DiPiero et al., 2023).

This special issue of Neurobiology of Language, titled The White Matter Connectome Supporting Speech and Language in the Human, responds directly to these challenges. The 10 contributions gathered here advance the field through empirical and methodological innovations, applying techniques such as restriction spectrum imaging (RSI), fixel-based analysis, and network control theory to dissociate specific fiber populations and their functional roles. Collectively, they move the field toward a comprehensive understanding of the white matter connectome for language—a framework that is dynamic, plastic, and clinically predictive.

We organize this overview into three thematic sections. First, Developmental and Experiential Plasticity examines how the connectome adapts (or remains stable) in the face of sleep, schooling disruptions, and prenatal exposures. Second, Lateralization and the Connectome reevaluates hemispheric dominance, moving beyond binary classifications to explore how interhemispheric transfer and intrahemispheric asymmetry jointly determine function. Third, Clinical and Disorder-Related Insights demonstrates how connectome integrity constrains recovery in aphasia and stroke, and how it characterizes neurodevelopmental conditions such as dyslexia. In what follows, we highlight how each paper contributes to these themes and charts the future of the field.

## DEVELOPMENTAL AND EXPERIENTIAL PLASTICITY

A recurring message of this issue is that white matter pathways supporting language are not static; they are malleable across development and sensitive to experience. Four studies in this issue underscore this point, spanning infancy through adulthood and incorporating both risk and resilience factors.

Curtis et al. (2025) provide a detailed examination of the structural development of speech networks in young children. First, they move beyond global tract averages by applying automated fiber quantification (AFQ) to derive nodewise, along-tract profiles, enabling detection of focal structure–behavior effects that would be obscured by mean diffusion metrics across an entire pathway. Using this approach, they highlight a lateralized role for the frontal aslant tract (FAT): children classified as higher versus lower performers on the Syllable Repetition Task showed robust differences along the left, but not right, FAT across contiguous nodes, consistent with a left-dominant speech–motor planning architecture. This AFQ result is important on its own because it localizes the effect to specific FAT segments rather than implying a uniform tract-wide association. It is especially compelling in light of the complementary whole-brain diffusion modeling with RSI. RSI separates hindered and restricted signal components and thus offers greater biological specificity than conventional tensor metrics, including sensitivity to diffusion signal differences in grey matter. In voxelwise tests, Curtis et al. (2025) report age-moderated relationships between speech accuracy and RSI indices in left inferior frontal regions (including *pars opercularis*) and white-matter territory traversed by the FAT, as well as parallel interactions within cerebellar white matter and adjacent grey-matter regions implicated in speech timing and sequencing. Taken together, the AFQ tract-profile sensitivity (left > right FAT) and the RSI evidence for age-dependent microstructural–behavior coupling, including convergent findings in cerebellar white and grey matter, point to the same mechanism. That is, the left FAT and its cortico–cerebellar partners constitute an early-developing scaffold for speech initiation and sequencing that strengthens measurably across the 4–7-year window, helping to explain why individual differences in speech accuracy track left-lateralized white-matter organization during early childhood (Curtis et al., 2025).

Ghasoub et al. (2025) extend the developmental focus to prenatal risk factors by examining toddlers exposed to *prenatal alcohol exposure* (PAE) in a South African birth cohort. Using diffusion MRI and tractography, the authors investigated associations between PAE, early white matter connectivity, and emergent language skills. In results that they appropriately interpret cautiously, toddlers with PAE showed higher clustering and local efficiency compared to unexposed peers. They also reported PAE moderated associations between network properties and Bayley Expressive Communication. These patterns are consistent with the idea that there are early alterations in network organization during a sensitive window. Methodological strengths they highlight include (a) a population-based South African birth cohort spanning Afrikaans- and isiXhosa-speaking families, (b) native-language, blinded administration of BSID-III expressive/receptive subtests, and (c) careful diffusion QC with natural-sleep scanning and strict motion exclusions that reduced the initial MRI sample to high-quality datasets. At the same time, the authors are explicit about limitations that qualify interpretation. The sample size is small (*n* = 23) due to the difficulty of recruiting such a sample. The sample also has prevalent prenatal tobacco exposure covariation, the design is cross-sectional, and there are potential socioeconomic influences that required inclusion of household income as a covariate. The cerebellum is also excluded from the AAL-based network, and several nominal PAE-by-network interactions did not survive multiple-comparison control. In their view, these data are preliminary and motivate longitudinal, multisite studies that integrate richer exposure phenotyping and multilingual context, and that test whether early “hyperconnectivity” (higher clustering/local efficiency) represents delayed pruning, compensatory reorganization, or measurement differences tied to toddler tractography thresholds. Overall, the paper advances the special issue’s developmental theme by placing PAE effects squarely in a connectomic framework while modeling the transparency needed when findings are uncorrected and effect sizes modest (Ghasoub et al., 2025).

Environmental disruptions later in development are also addressed in this issue. Blockmans et al. (2025) study the impact of COVID-19 school closures on white matter plasticity in the reading network in children in early primary school. Using longitudinal diffusion data collected before and after pandemic-related closures, they test whether interruptions in formal instruction affected reading-related tracts, including the arcuate fasciculus and inferior longitudinal fasciculus. Behavioral results revealed modest short-term setbacks in phonological awareness, but white matter indices remained stable across the disruption. This suggests both resilience of the reading connectome and the possibility that white matter properties change more slowly than acute environmental conditions. The study also raises critical questions: Are there sensitive windows where environmental stressors do leave lasting traces? Are some tracts more susceptible than others? This paper will spark debate on the relative stability versus plasticity of language-related white matter. The two-cohort design (pre-closure vs. closure cohorts; *N* = 160 first-graders, many at cognitive risk of dyslexia) did not show evidence 1 year after closure of altered fractional anisotropy (FA) in canonical reading tracts. FA increased over time at comparable rates in the arcuate fasciculus and inferior fronto-occipital fasciculus, with converging nulls in the inferior longitudinal fasciculus. Neither parental support nor closure duration explained individual differences in tract growth, and the null cohort by time interaction persisted when restricting analyses to the most vulnerable subsample, and when imputing missing tract data. Methodologically, Blockmans et al. (2025) emphasize (a) strict motion handling and nodewise along-tract analyses (arguing against overreliance on tract-mean FA), and (b) limitations of deterministic AFQ tracking in young children (e.g., frequent right-AF dropouts), advocating future use of constrained spherical deconvolution (CSD)-based probabilistic pipelines and multimodal indices (e.g., mean diffusivity [MD], myelin-sensitive measures) to test whether different tissue markers might be more sensitive to short-lived educational disruptions. Their Discussion frames the overall pattern as reassuring for the reading connectome in this high-income, Dutch-speaking context with relatively brief first-grade closures, while calling for cross-site replications in more heterogeneous settings, designs that might capture *immediate* post-closure neural effects, and analyses that probe whether stress-sensitive networks outside the canonical reading circuitry may show stronger pandemic-related signatures (Blockmans et al., 2025).

Finally, Earle et al. (2025) offer a novel perspective by linking white matter to *sleep-dependent consolidation* of new phonetic categories in adults. Participants were trained to discriminate non-native speech sounds and tested before and after a night of sleep, situating their interpretation within a complementary learning systems account in which successful category learning shows a post-sleep shift from episode-bound representations toward a more abstract cortical code. Consistent with this, Day-2 scans (after sleep) showed greater engagement of cortical speech regions (with the addition of left insula) relative to the immediate post-training session, suggesting an overnight transfer toward speech-selective cortex. Importantly, individual differences in white-matter microstructure predicted who benefited most. FA in the anterior thalamic radiation and the uncinate fasciculus was associated with the magnitude of overnight improvement, especially for generalization to an untrained talker, and even after controlling for initial learning and actigraphy-estimated sleep duration. Beyond this, the study bridges the white matter and language learning literature with sleep and memory consolidation, highlighting how experience interacts with fronto-temporal connectivity to shape language outcomes. The Discussion also clarifies important boundary conditions. That is, consolidation benefits for identification tasks tend to be more robust than for fine-grained discrimination under high-variability test conditions (e.g., an untrained talker), an asymmetry that helps interpret the mixed overnight discrimination results here. Methodologically, the authors are transparent about limits that temper inference, especially the small, all-female sample. However, despite these caveats, the results suggest that sleep appears to reorganize reliance from hippocampal and episode-bound traces toward cortical speech systems, while consolidation capacity is scaffolded by specific white-matter pathways. Future work should combine structural predictors with sleep physiology (e.g., spindle dynamics) to advance a mechanistic understanding and to test whether ventral fronto-temporal connectivity plays a privileged role in cross-talker generalization (Earle et al., 2025).

Together, these four contributions emphasize that the white matter connectome is a changing system—it develops rapidly in childhood, is shaped by prenatal exposures and schooling environments, and constrains the consolidation of new learning in adulthood. These studies collectively reinforce the principle that white matter is not inert scaffolding but a substrate of ongoing developmental and experiential plasticity.

## LATERALIZATION AND THE CONNECTOME

Why is language so often left-lateralized, and what structural features predict variability in this lateralization? Two papers in this issue tackle this foundational question, bringing complementary approaches to a long-standing debate.

Zhu et al. (2025) sharpen the lateralization story by separating two questions: (a) which white-matter features differ between language-dominance groups (categorical left- vs. right-dominant) and (b) which features vary with the continuous degree of lateralization. They address both questions in *n* = 96 left-handers, who have a higher prevalence of atypical dominance, thereby providing stronger leverage on structure–function links. Using a fixel-based framework (fiber-specific metrics), they report that group differences for language dominance localize to asymmetry of the superior longitudinal fasciculus III (SLF III), whereas group differences for spatial-attention dominance localize to interhemispheric pathways, specifically the rostrum and rostral body of the corpus callosum. Importantly, the direction of functional lateralization did not mirror the direction of tract asymmetry, arguing against a simple “more leftward tract → more left-lateralized function” mapping. Instead, continuous variation in language lateralization was predicted by a combined model including SLF-III asymmetry (intrahemispheric) and the rostral callosal body (interhemispheric), while the degree of spatial-attention lateralization was predicted by the callosal rostrum alone. In all analyses, stronger functional lateralization was negatively related to anterior/middle callosal connectivity, consistent with an excitatory callosal account in which denser interhemispheric coupling reduces hemispheric differences by promoting cross-talk (as opposed to an inhibitory model). In their discussion, the authors emphasize three implications of these results: (a) lateralization is better explained by the balance of intra- and interhemispheric connections than by tract-direction asymmetry per se; (b) language and spatial attention rely on distinct structural substrates (SLF-III vs. anterior corpus callosum); and (c) sampling left-handers is essential to expose these relationships that are hard to see in overwhelmingly typical right-handed cohorts. They are also explicit about limits to these interpretations, including an all left-handed sample, and the fact that lateralization phenotypes are defined by a single task, both of which that constrain generalizability (Zhu et al., 2025).

Desjardins et al. (2025) take a different tack, leveraging the Human Connectome Project’s large sample of over 600 participants. They test whether microstructural and macrostructural properties of key tracts—the arcuate fasciculus, inferior longitudinal fasciculus, frontal aslant tract, and corpus callosum—predict functional lateralization indices derived from resting-state fMRI. Concretely, they computed a seed-based resting-state language lateralization index and paired it with tractography-derived features from both hemispheres, then evaluated linear-regression and random-forest models. Several tract features emerged as statistically reliable correlates of the lateralization index, but out-of-sample predictive performance was low, indicating that current structural indicators are not sufficiently representative to act as practical proxies for functional lateralization. They outline likely reasons, namely that lateralization index here is resting-state and seed-defined, limiting generalizability. Moreover, the rs-fMRI approach assumes stationarity across long acquisitions, potentially masking meaningful temporal dynamics. The constructive takeaway is forward-looking. The authors argue that moving toward a clinically useful “structural lateralization index” will likely require richer, explicitly multimodal models that integrate fiber-specific measures with dynamic functional connectivity. In short, the study narrows the candidate space, clarifies methodological boundaries, and sets a benchmark for predictive work on the connectome basis of language lateralization (Desjardins et al., 2025).

Together, these contributions shift the discussion of lateralization from a binary phenomenon (“left versus right”) toward a measurable, variable property embedded in the connectome. They remind us that lateralization is not a simple categorical trait but a probabilistic outcome shaped by a complex interplay of intra- and interhemispheric connectivity. This perspective opens new possibilities for understanding individual differences, atypical lateralization, and their consequences for language function.

## CLINICAL AND DISORDER-RELATED INSIGHTS

A third set of contributions demonstrates how connectome science can shed light on disorders of language and their recovery. These studies extend the white matter connectome framework to aphasia, working memory, dyslexia, and writing, showing how structural connectivity informs both deficits and compensation.

Stoll et al. (2025) use network control theory to test whether the right *pars triangularis* takes on a different structural role after left-hemisphere stroke. They estimated *boundary controllability* from diffusion-based structural connectomes in 60 people with aphasia and 62 controls. Two results anchor their Discussion. First, right *pars triangularis* controllability was higher in the aphasia group. Second, within the aphasia group, higher right *pars triangularis* controllability predicted fewer phonological naming errors. The authors argue that this helps reconcile mixed findings about right inferior frontal involvement in recovery. What matters may be the node’s capacity to flexibly link systems that support naming, not its activation level alone. This connects the right inferior frontal gyrus to phonological control in a way that is measurable in the connectome (Stoll et al., 2025).

They also note important limits. The design is cross-sectional, so changes over time are unknown. Furthermore, controllability is inferred from diffusion data and depends on tractography choices. In addition, lesions varied across patients, which can reshape interhemispheric pathways differently. Finally, the study did not pair controllability with functional measures during language tasks. The authors close by calling for longitudinal, multimodal work that combines diffusion with task or resting fMRI/MEG, and for state-dependent neuromodulation studies. These steps could test whether right inferior frontal gyrus controllability can be adjusted to improve phonological recovery and identify who is most likely to benefit (Stoll et al., 2025).

Martin et al. (2025) tackle whether recovery of domain-specific verbal working memory depends on left- or right-hemisphere tracts after left-hemisphere stroke. In a longitudinal design with 24 patients tested acutely (within ∼4 days) and again >6 months later, only semantic, but not phonological working memory, improved (Martin et al., 2025). Gains in semantic working memory correlated with left-hemisphere FA in the direct segment of the arcuate fasciculus, inferior fronto-occipital fasciculus, and inferior longitudinal fasciculus, and these relations survived false-discovery–rate correction (Martin et al., 2025). No right-hemisphere tract predicted semantic working memory recovery, and neither hemisphere’s tracts predicted phonological working memory change. The Discussion situates these findings against a literature that often emphasizes grey-matter reorganization and sometimes implicates right-hemisphere support. Here, white-matter predictors are left-lateralized for semantic working memory recovery, sharpening debates about the conditions under which right-hemisphere recruitment is beneficial (Martin et al., 2025).

Martin and colleagues also probe alternative explanations. The null finding for phonological working memory is *not* due to restricted variability. Phonological change scores were as variable as, or slightly more variable than, semantic change scores, so simple power or range limitations are unlikely to explain the absence of tract–behavior links (Martin et al., 2025). Instead, the authors argue that semantic working memory recovery may depend more directly on the integrity of long-range occipito-temporal and fronto-temporal connections, consistent with prior work linking semantic working memory to sentence comprehension and to left-lateralized pathways (Martin et al., 2025). The take-home message from their Discussion is conservative and clinically relevant. Structural connectivity in left arcuate fasciculus, inferior fronto-occipital fasciculus, and inferior longitudinal fasciculus supports the recovery trajectory of semantic working memory, while right-hemisphere tracts do not appear to compensate for this component. Future work should add dynamic functional measures and larger samples to test whether targeted therapy or neuromodulation that strengthens left-hemisphere pathway function yields better semantic working memory outcomes (Martin et al., 2025).

Verhelst et al. (2025) revisit the long-standing claim that dyslexia shows robust white-matter alterations. Using fixel-based analysis in 35 adults with dyslexia and 34 matched controls, they report no group differences at whole-brain, tract-specific, or tract-averaged levels (Verhelst et al., 2025). Yet, within the dyslexia group, poorer word reading related to higher fiber density and cross-section in bilateral inferior longitudinal fasciculus. The Discussion leans into this tension. First, the absence of between-group effects in adults suggests that white-matter differences may be smaller, heterogeneous, or time-limited by adulthood. Second, the inferior longitudinal fasciculus correlation is surprising in its direction. That is, higher cross-section typically implies greater capacity, prompting hypotheses about maladaptive overreliance on visual–orthographic pathways when more efficient compensatory routes fail to develop (Verhelst et al., 2025).

The authors also clarify an analytic nuance regarding tract-averaged fiber density and cross-section. This measure captured the inferior longitudinal fasciculus–reading association even when only a handful of fixels reached significance in tract-specific tests. They argue this pattern fits the idea that dyslexia-related effects may be spatially variable across individuals along the same tract, favoring an approach that combines fixel-level and tract-averaged analyses in the same study (Verhelst et al., 2025). Their Discussion explicitly calls for larger, pooled datasets and harmonized pipelines to adjudicate small effects and developmental timing questions, while noting that task-based functional differences in dyslexia are well established even when macrostructural white-matter differences are not (Verhelst et al., 2025). They argue that adult dyslexia may not show reliable *group* alterations in white matter, but links to reading within specific tracts (inferior longitudinal fasciculus) remain informative about how reading skill varies, even if the mechanistic directionality (beneficial vs. compensatory/maladaptive) requires longitudinal testing.

Sagi et al. (2025) extend examination of the connectome to central processes in written word production. In 73 neurotypical adults performing a spelling-to-dictation task, they reconstructed bilateral cerebello–thalamo–cortical pathways (CTCs) and the frontal aslant tract using probabilistic tractography, then correlated along-tract FA with spelling accuracy. The key finding is selective: spelling accuracy correlates with FA in the left CTC (left cerebellar hemisphere to right cerebrum), with a trend on the right, and this holds even after controlling for spoken production measures. In contrast, frontal aslant FA does not correlate with spelling (Sagi et al., 2025). In the Discussion, the authors interpret this as the first direct evidence that cerebello–cerebral connectivity contributes to central orthographic processes, consistent with CTC projections to motor, prefrontal, parietal, and temporal association areas and with growing recognition of cerebellar roles in higher level language (Sagi et al., 2025).

They further argue that these results broaden a literature focused on “classical” dorsal/ventral language pathways. Prior diffusion work linked spelling to inferior longitudinal fasciculus, uncinate fasciculus, and arcuate fasciculus, but Sagi et al. show that CTCs add unique variance and that the FAT may be more relevant for speech fluency than for central spelling (Sagi et al., 2025). Methodological strengths include individual-subject probabilistic tracking and along-tract profiling. Their Discussion also lays out clear next steps: (a) delineate which *specific* spelling subprocesses (e.g., phoneme–grapheme conversion vs. lexical orthography retrieval) engage CTC; (b) test whether CTC relates to orthographic working memory buffers, and (c) examine whether FAT involvement emerges for peripheral output demands (e.g., handwriting/typing) rather than central mapping. By locating spelling within cortico–cerebellar loops, the paper situates orthographic production within a distributed network that extends beyond the Perisylvian cortex (Sagi et al., 2025).

Together, these clinical and disorder-focused contributions illustrate how connectome science is beginning to inform intervention, prognosis, and theoretical models of language disorders. They also show how new methods, including network controllability, fixel-based analysis, and tract-specific profiling, are reshaping what we know about structure–function relationships in clinical populations.

## CONCLUSION

This special issue advances the field of neurobiology of language in several key ways. It demonstrates that the white matter connectome is (a) *dynamic*, changing across development and in response to environmental experience; (b) *variable*, with asymmetries and lateralization shaped by structural features; and (c) *clinically relevant*, informing our understanding of disorders from dyslexia to aphasia. Methodologically, it also raises the bar, employing advanced diffusion models, tract profiling, connectomics, and large datasets.

The collective message is forward-looking: the white matter connectome is not merely scaffolding for cortical computation but an active, plastic, and clinically important substrate of speech and language. Future research will build on these foundations by integrating structural and functional measures, leveraging longitudinal and large-scale datasets, and applying connectome science to intervention. This special issue positions the field for this next stage, moving beyond tract-specific localization toward a network-level, dynamic, and translational science of language.
